# Web-Based Relaxation Intervention for Stress During Social Isolation: Randomized Controlled Trial

**DOI:** 10.2196/22757

**Published:** 2020-12-03

**Authors:** Silvia Francesca Maria Pizzoli, Chiara Marzorati, Davide Mazzoni, Gabriella Pravettoni

**Affiliations:** 1 University of Milan Department of Oncology and Hematology-Oncology Milan Italy; 2 Applied Research Division for Cognitive and Psychological Science European Institute of Oncology IEO, IRCCS Milan Italy

**Keywords:** relaxation, guided meditation, web-based intervention, social isolation, intervention, COVID-19, anxiety, stress, internet

## Abstract

**Background:**

Relaxation practices might be helpful exercises for coping with anxiety and stressful sensations. They may be of particular utility when used in web-based interventions during periods of social isolation.

**Objective:**

This randomized study aimed to test whether web-based relaxation practices like natural sounds, deep respiration, and body scans can promote relaxation and a positive emotional state, and reduce psychomotor activation and preoccupation related to the COVID-19 pandemic.

**Methods:**

Participants were randomly assigned to one of three experimental conditions. Each condition was characterized by a single online session of a guided square breathing exercise, a guided body scan exercise, or natural sounds. The participants listened to one of the fully automated audio clips for 7 minutes and pre-post completed self-assessed scales on perceived relaxation, psychomotor activation, level of preoccupation associated with COVID-19, and emotional state. At the end of the session, qualitative reports on subjective experience were also collected.

**Results:**

Overall, 294 participants completed 75% of the survey and 240 completed the entire survey as well as one of three randomly assigned interventions. Perceived relaxation, psychomotor activation/stress, and preoccupation related to COVID-19 showed a positive improvement after participants listened to the audio clips. The same pattern was observed for the valence and perceived dominance of the emotional state. The square breathing and body scan exercises yielded superior results compared to natural sounds in lowering perceived stress.

**Conclusions:**

This study provides a novel insight that can guide the development of future low-cost web-based interventions to reduce preoccupation and stress in the general population.

**International Registered Report Identifier (IRRID):**

RR2-10.2196/19236

## Introduction

### Background

To limit the spread of the SARS-CoV-2 virus, from mid-March to May 2020, Italy faced a strict lockdown [1]. People were forced to form a bubble with only members of their household, if they had any. This social isolation and resulting loneliness had an impact on health and mental well-being, thus affecting vital functions (eg, sleep quality), social connectedness, perceived support, and psychological status [2-4]. A recent rapid review of the effects of quarantine highlighted that many people were facing several negative cognitive and emotional problems, like confusion, poor concentration, irritability, insomnia, distress, frustration, and anger [5]. People were worried about the quarantine duration, insufficient information provision, economic problems, and stigma. The associated negative effects had an impact on biopsychosocial functioning, sometimes even leading to depressive or posttraumatic stress symptoms [5,6].

The lack of a vaccine, the high chance of contagion, and the severity of symptoms—sometimes resulting in death—increased risk perception. In all high-risk situations, cognitive and rational thinking interacts with emotional appraisals, thus affecting people's state of mind: individuals feel vulnerable and may experience fear for themselves and for their loved ones [7,8]. Both physical and psychological dimensions are affected by the sense of uncertainty and the threat of contracting the virus. In a similar emergency condition, it is not uncommon that people may also experience a psychophysiological hyperactivation, thus paying excessive attention to bodily sensations and enhancing their perceptions [9]. Overall, COVID-19 and social isolation have led to negative side effects, causing widespread concern and psychophysiological reactions [5].

Starting from these premises, it appeared of primary importance to develop efficacious interventions aimed at reducing the possible preoccupations about COVID-19 and the associated psychophysiological activation.

In this regard, previous studies demonstrated that interventions based on natural sounds, respiration, and meditation helped individuals to alleviate the effects of stress by reducing physiologic arousal and restoring autonomic balance [10-13].

In fact, listening to natural sounds significantly reduces human stress processes [12-14]. For example, the stress recovery theory [15] posits that physiological (autonomic) and psychological stress are reduced within naturalistic environmental contexts because of human evolutionary adaptation to naturalistic stimuli. On the other hand, guided relaxation techniques represent widely used practices to produce a deep state of relaxation and enhance physical and emotional well-being. Deep breathing exercises and focusing attention on body perception (body scan) are two of the main techniques to reduce hyperarousal and achieve a more relaxed condition. The former may also be defined as “an efficient integrative body-mind training for dealing with stress, anxiety and psychosomatic conditions” [16]; it may help people slow their breathing, take in more oxygen, and reduce the use of shoulder, neck, and upper chest muscles, thus achieving better emotional balance and social adaptation [17]. On the other hand, body scans aim to focus attention on different parts of the body and encourage awareness of the body's sensations, such as pain, tension, warmth, or relaxation [18,19]. Applying these interventions to people who are forced into mandatory social isolation may help people become more aware of their mind-body condition and reduce negative effects.

We must add that, due to COVID-19 restrictions, face-to-face interventions were not possible, while web-based interventions represented an important opportunity. The usefulness of web-based interventions is supported by studies showing that online relaxation interventions led to significant results equal to in-person interventions [20,21]. However, a comparison of meditation techniques (eg, natural sounds, respiration, and body scan) in web-based interventions is still missing in the literature.

We chose to employ simple audio clips remotely delivered with auditive natural stimuli (water sounds), which already proved to be effective in facilitating the relieving of psychophysiological activation linked to a psychological stressor [14], along with two exercises with guiding instructions targeting body awareness and breath frequency control.

### Objective

In this study, we aimed to test and compare the efficacy of three web-based interventions (based on natural sounds, breathing regulation, and body scan, respectively) to assess which intervention was the most effective for the target population. For this purpose, we tested the differences in the stress-reducing efficacy of three audio clips corresponding to the three relaxation practices (square breathing exercise, guided body scan exercise, and natural sounds). Specifically, we expected to find the following: (1) a decrease in the levels of psychomotor activation/stress and of preoccupation about COVID-19, as well as enhanced levels of relaxation and emotional state after exposure to all audio clips, and (2) that guided techniques (square breathing and body scan) would have a greater effect than natural sounds on the abovementioned dimensions.

## Methods

### Participants and Procedure

During the first week of May 2020, the invitation to take part in the study was published on Italian social media webpages (specifically, organic posts on WhatsApp, Facebook, LinkedIn, and Instagram). The readers were informed about the general aim of the study and that the study was conducted by researchers from the University of Milan. Potential participants were encouraged both to take part in the study and to share the invitation with their acquaintances. The invitation contained a link to the Qualtrics platform, where a more detailed description was available.

The eligibility criteria to take part in the study were the following: (1) being older than 18 years old, (2) being a proficient Italian speaker, (3) not having any impairment of auditory abilities, and (4) having an appropriate familiarity with computer literacy. Before taking part in the study, participants were asked to read and complete an online consent form.

Participation in the study consisted of three main parts: (1) a short questionnaire containing sociodemographic questions, a baseline anxiety evaluation (trait anxiety, anxiety for physical sensations, body vigilance), and a preintervention evaluation, (2) listening to a 7-minute audio clip, and (3) the postintervention evaluation. The estimated time for participating in the study (completing the three parts) ranged from 12-17 minutes.

Participation in the study was voluntary and participants were informed that they could withdraw from the study at any point in time. The research protocol followed the CONSORT-EHEALTH (Consolidated Standards of Reporting Trials) V1.6 Guidelines [22] and the principles stated in the Declaration of Helsinki (59th WMA General Assembly, Seoul, 2008). The research protocol was approved by the Ethics Committee of the first author’s university on April 30, 2020, and registered with the following International Registered Report Identifier (IRRID): PRR1-10.2196/19236 [23].

Overall, 294 participants completed at least 75% of the survey. The initial sample was mainly composed of female participants (women: n=216, 73.5%; men: n=78, 26.5%) and participants had a mean age of 39 years (SD 14.6, range 18-78). Overall, 87 (29.6%) of the participants who completed at least 75% of the survey had a chronic disease condition, while 207 (70.4%) had no chronic health issues. Participants were also asked to specify which chronic disease they have. The specific chronic conditions were organized into categories and are reported in Multimedia Appendix 1. The three initial groups were homogeneous in sociodemographic variables like gender (*χ*^2^_2_=.91, *P*=.63), age (*F*[2,291]=1.59, *P*=.21), educational level (*χ*^2^_10_=5.4, *P*=.86), marital status (*χ*^2^_6_=1.14, *P*=.98), and employment status (*χ*^2^_6_=3.5, *P*=.73).

### Measures and Design

#### Baseline Questionnaire

After completing the sociodemographic form, participants were asked to report if they had a chronic disease and how much the disease impacted their (perceived) vulnerability to COVID-19. Participants were asked to report their working situation, recent changes in occupational status due to COVID-19 restrictions, and if they had prior experience with relaxation techniques.

Participants were then asked to complete 3 self-assessed questionnaires aimed at measuring their current level of anxiety (trait anxiety), tendency to worry about physical signals and sensations (anxiety for physical sensations), and degree of attention paid to bodily feelings (body vigilance). For the assessment of these aspects, the following self-reported scales were used: the State-Trait Anxiety Inventory form-trait subscale (STAI-Y) [24,25], the Physical Concerns subscale of the Anxiety Sensitivity Index-3 (ASI-3) [26,27], and the Body Vigilance Scale (BVS) [28].

The STAI-Y is a questionnaire of 20 items scored on a 4-point Likert scale (from 1=not at all to 4=very much) [24,25], and assesses trait anxiety. The total score is calculated as the sum of all the items, after having computed the reverse scores of specific items. A higher total score indicates higher anxiety. The STAI-Y has a good internal consistency and it constitutes a reliable and valid tool for assessing anxiety symptoms in samples of healthy subjects [29]. In our sample, the Cronbach α was .84, indicating a good level of internal consistency.

The Physical Concerns subscale of the ASI-3 is a 6-item subscale and participants use a 5-point Likert scale (from 0=very little to 4=very much) to indicate worry related to specific physical sensations. The total score is the sum of the single items [30]. In our study, the Physical Concerns subscale had a high Cronbach α of .88, showing a good level of internal consistency.

Finally, the BVS is 4-item questionnaire (rated on a scale from 0=not at all like me to 10=completely like me) that asks how much attention one usually pays to body sensations. In the fourth item, participants had to rate their attention to 15 body sensations that are the core physical symptoms for panic attacks [31]. In this study, the internal consistency was .86.

#### Randomization Procedure

After completing questionnaires, participants were randomly assigned to one of the three experimental groups via the randomization procedure within Qualtrics. The randomization option was set to enroll the same number of subjects for each condition.

In each experimental condition, participants received a 7-minute audio clip aimed at promoting a state of awareness and relaxation. In the first experimental condition (square breathing), participants heard a recorded voice guiding the regulation of breathing frequency, with the aim of making every breath cycle (inhalation, hold breath, exhalation, hold breath) the same length (4 seconds). In the second experimental condition (body scan), participants listened to an audio clip with a voice that guided participant attention through every part of the body and gently requested that the listener notice and let go of tensions and unpleasant feelings. Both tracks were recorded by a trained mindfulness and yoga expert in collaboration with a psychotherapist and were pretested on 4 subjects to assess how easy the exercise is and its perceived effectiveness. In the final condition (natural sounds), participants were presented with a prerecorded audio clip of natural sounds (rain, water sounds). The scientific literature indicates that exposure to natural sounds may be an important stress reliever, thus promoting a state of relaxation [12,14].

All audio clips were preceded by instructions regarding the recommended location and body position for the exercises. More precisely, participants were invited to find a quiet room and to sit or lie down on a comfortable chair/sofa. After the instructions, participants were invited to click on the “play” key to start the audio clip.

#### Pre-Post Evaluation

As pre-post measures, before and after the audio stimuli, subjects were asked to self-rate their perceived relaxation level, perceived stress, and psychomotor activation degree (ie, motor/physical activity that is secondary to or dependent upon a psychic component and is mostly non–goal-directed [32]), how much they felt concerned about COVID-19, and to rate 3 specific features of their emotional state. Specifically, participants were requested to rate on 3 Visual Analogue Scales (VAS; 0=not at all, 10=completely) how relaxed they felt, how psychomotor-activated they felt, and how much thoughts related to COVID-19 scared them; furthermore, they completed the Self-Assessment Manikin (SAM) [33] for emotional states, which is a 3-item visual and nonverbal scale. Valance (from “unpleasurable” to “pleasurable”), intensity/arousal (from “calm” to “excited”), and dominance (from “not in control” to “completely in control” of emotional state) are commonly used to quantify properties of the felt overall emotional state on 1-5 scales of images. To check if participants really listened to the audio clips, they were asked immediately after the recording if they heard the entire clip, a part of it, or nothing.

Finally, all the participants were asked to describe their personal experience and to provide suggestions for future changes. Specifically, participants were requested to write a short paragraph answering two open-ended questions about their personal experience with the exercise (ie, what they liked and what they would have changed).

### Data Analysis

A detailed report of the data analysis approach can be found in [23]. Compared to the original protocol, we had a slightly lower sample size, which yielded a statistical power of .94 instead of the planned .95, for a medium effect size (ES). To reduce the chance of type I error, we choose to test and compare the efficacy of the audio clips with the mixed analysis of variance (ANOVA) and to report ES in the form of *η*^2^; we also performed post hoc comparisons with the Tukey HSD test, instead of performing multiple comparisons with the *t* test.

To test the difference in efficacy between audio clips, a one-way ANOVA on gain relaxation scores, with 3 groups and fixed effects, and no interaction, was performed. Subjects who stopped before the randomized exposure to the audio clips were excluded from this analysis. Furthermore, to assess group and time effects and their interaction, a 2 (time) × 3 (groups) mixed-model ANOVA was performed for perceived relaxation scores, perceived stress/activation, and preoccupations related to COVID-19. We also performed nonparametric analysis on the items of SAM, as statistical assumptions for parametrical analysis were violated. In this case, we reported the ES in the form of Hodges-Lehmann ES.

Explorative analyses on the possible role of trait anxiety, anxiety for physical sensations, and body vigilance in moderating the effect of the audio clips were also carried out. All quantitative analyses were performed in SPSS (Version 26.0; IBM Corp).

Qualitative reports on subjective experiences were organized into different categories, to systematize the suggestions and preferences. Participants’ preferences on the web-based relaxation interventions were organized into three different categories (audio features, relaxing feeling, and awareness) to systematize participants’ experience. Finally, the reported suggestions for improving the quality of the interventions were grouped into three main themes: audio features, clarity of instructions, and length of the intervention.

## Results

### Sample Description

Overall, 328 participants registered on the survey website, and 294 gave written informed consent and completed more than 75% of the survey (meaning that they complete the initial questionnaire and were randomized into the three conditions), but they did not answer the postexposure questionnaire. Further, 12 of the 294 participants also stated that they did not listen to the audio clips. In total, 240 participants completed the entire survey and stated that they listened to the audio clips: 77 in square breathing group, 76 in the body scan group, and 87 in the natural sounds group. Among those who completed the entire survey (240), the mean age was 39.8 (SD 14.7, range 18-78) years, while 173 (72.1%) were female and 67 (27.9%) were male.

In regard to the differences in baseline variables (age, sex) between those who completed the entire survey and those who did not, no differences were found to be significant. Specifically, no differences emerged in gender (*χ^2^*_1_=1.28, *P*=.26) and age (*F*[1,292]=1.1, *P*=.87).

In total, 70 (29.2%) participants had a chronic disease condition, while 170 (70.8%) had no chronic health issues. In addition, 15 (6.3%) reported to be highly limited in daily activities because of a disease, while 26 (10.8%) were partially influenced by health issues and 97 (40.4%) were not affected in daily activities by health issues. With regard to the differences between participants who completed the entire survey and those who did not, no differences emerged with the presence of a chronic disease (*χ*^2^_1_=1.11, *P*=.74).

In regard to risk perception related to COVID-19, we found that participants perceived a small amount of risk of contracting COVID-19 (median 2=a little bit) and of having serious side effects of COVID-19 (median 2=a little bit). Participants reported being moderately worried about their employment status, health, and personal economic stability (median 3=quite a lot), while they were more preoccupied for their own family (median 4=a lot preoccupied). In regard to previous experience with relaxation practices, 101 participants (42.1%) had previous experience with relaxation techniques, while 139 (57.9%) had no previous experience with relaxation practices. Descriptive statistics of the final sample are reported in Table 1.

**Table 1:** Descriptive statistics of the sample.

Sociodemographic variables		Frequency, n (%)
**Educational level (N=240)**		
	Middle school diploma	12 (5.0)
	High school diploma	87 (36.3)
	Bachelor’s degree	33 (13.8)
	Master’s degree	78 (32.5)
	Postgraduate	30 (12.5)
**Marital status (N=240)**		
	Single	68 (28.3)
	In a relationship	62 (25.8)
	Married or cohabitating	107 (44.6)
	Widowed	3 (1.3)
**Employment status (N=240)**		
	I work	184 (76.7)
	I do not work and I am not seeking work	42 (17.5)
	I do not work but I am seeking work	14 (5.8)
**Work contract (N=228)**		
	Temporary	38 (16.7)
	Permanent	110 (48.2)
	Freelance	59 (25.9)
	Other	21 (9.2)
**Work activity in the last week (N=228)**		
	Regular job activity	82 (36.0)
	Less than regular job activity	50 (21.9)
	No activity	75 (32.9)
	Other	21 (9.2)

### Trait Anxiety, Anxiety for Physical Sensations, and Body Vigilance

Descriptive statistics and reciprocal correlations of trait anxiety, anxiety for physical sensations, and body vigilance are provided in Tables 2 and 3.

The three scores correlated moderately with each other, consistent with what has been found in previous literature [29,30,34]. Furthermore, there were small to moderate positive correlations with postexposure psychomotor activation/stress and preoccupation related to COVID-19. Finally, small negative correlations were found between trait anxiety and anxiety for physical sensations, and postexposure perceived relaxation.

**Table 2:** Descriptive statistics of the total scores of the questionnaire scores.

Questionnaire	Mean (SD)	Median	Minimum-maximum	Correlation between total scores		
				STAI-Y^a^	ASI-3^b^	BVS^c^
STAI-Y	44. 5 (8.2)	43.5	28-68	1	.422^d^	.343^d^
ASI-3	18.3 (7.9)	18.5	0.3-39.7	N/A^e^	1	.517^d^
BVS	11.4 (4.4)	10.5	6-30	N/A	N/A	1

^a^STAI-Y: State-Trait Anxiety Inventory form-trait subscale.

^b^ASI-3: Anxiety Sensitivity Index-3.

^c^BVS: Body Vigilance Scale.

^d^*P*<.001.

^e^N/A: not applicable.

**Table 3:** Descriptive statistics of the correlations between total scores.

Variables	Correlation with postexposure measures		
	STAI-Y^a^	Anxiety Sensitivity Index-3	Body Vigilance Scale
Relaxation	–0.23^b^	–0.13^c^	0.1
Psychomotor activation/stress	0.48^b^	0.38^c^	0.3^b^
Fear related to COVID-19	0.19^b^	0.35^b^	0.21^b^

^a^STAI-Y: State-Trait Anxiety Inventory form-trait subscale.

^b^*P*<.001.

^c^*P*<.05.

For all three scales, higher scores indicated a higher presence of anxiety; overall, we found participants to have higher scores compared to the reference values.

Specifically, for both men and women (men: mean 43.1, SD 7.9; women: mean 45, SD 8.3), the total score on the STAI-Y in our sample was slightly higher than the means of the reference values (men: mean 36, SD 9.7; women: mean 39.93, SD 11) [35].

In terms of the ASI-3 Physical Concerns subscale in our sample (men: mean 10.1, SD 7.9; women: mean 18.7, SD 7.8), we found participants had higher levels of anxiety for physical sensations compared to the reference values (men: mean 4.99, SD 4.28; women: mean 5.91, SD 4.78) [30]. Finally, for the BVS (men: mean 17.4, SD 7.9; women: mean 18.7, SD 7.8), participants reported higher body vigilance levels compared to the normative sample (men: mean 14.86, SD 6.92; women: mean 15.95, SD 9.71) [34].

The mean scores of the questionnaires were homogeneous between those with a chronic disease and those with no health issues, except for the ASI-3 scores (*t*[238]=–2.6, *P*=.01); specifically, the participants with a chronic condition had significantly higher anxiety for physical sensations (mean 12.5, SD 5) compared to those who did not have a chronic condition (mean 10.9, SD 3.9).

Furthermore, based on the total scores for each experimental group, we found that that the three groups did not significantly differ in the initial scores of trait anxiety (*F*[2,237]=.02, *P*=.98), anxiety for physical sensations (*F*[2,237]=.09, *P*=.92), and body vigilance levels (*F*[2,237]=.17, *P*=.85).

Regarding differences between the initial sample and the final sample in psychological variables, we found no significant differences in the levels of trait anxiety (*F*[2,292]=1.2, *P*=.28), anxiety for physical sensations (*F*[2,292]=.83, *P*=.36), and body vigilance levels (*F*[2,284]=.25, *P*=.62).

### Perceived Relaxation, Psychomotor Activation/Stress, and Thoughts Related to COVID-19

Descriptive statistics of all the assessed pre-post variables for each group are depicted in Table 4.

**Table 4:** Descriptive statistics of assessed pre-post variables for each group.

Variables	Square breathing (N=77)		Body scan (N=76)		Natural sounds (N=87)	
	Pre	Post	Pre	Post	Pre	Post
Perceived relaxation, mean (SD)	47.7 (23.7)	65.9 (20.4)	47.4 (23.1)	64.8 (23.9)	44.7 (25.6)	60 (23.8)
Psychomotor activation/stress, mean (SD)	48.5 (25.3)	31 (22.7)	49.8 (26.2)	31.4 (22.7)	57.4 (25.5)	38.9 (26.9)
Thoughts related to COVID-19, mean (SD)	62.6 (26.6)	47.5 (27.8)	64.6 (26.9)	51.4 (28)	66.6 (25.3)	54.3 (27)
Valence, median (IQR)	5 (2)	7 (2)	5 (4)	7 (3)	5 (4)	6 (3)
Arousal, median (IQR)	4 (2)	5 (4)	5 (3)	5 (3)	4 (3)	4 (3)
Dominance, median (IQR)	6 (3)	7 (2)	5 (3)	6 (2)	6 (2)	7 (2)

Age was slightly positively correlated with fears associated with COVID-19 (*r*=.21, *P*=.001) and with ASI-3 scores (*r*=.25, *P*=.001).

As a primary analysis to compare efficacy between the audio clips, we performed a one-way ANOVA on postexposure relaxation scores. The analysis yielded nonsignificant differences between groups in perceived relaxation after exposure to the relaxing audio clips (*F*[2,237]=1.6, *P*=.21).

To test and compare the efficacy between the audio clips, a mixed ANOVA testing for group and time effects and for their interaction was performed for perceived relaxation scores, perceived stress/activation, and preoccupations related to COVID-19.

All effects reported below are stated as significant at *P*<.001 unless otherwise specified.

**Figure 1:**
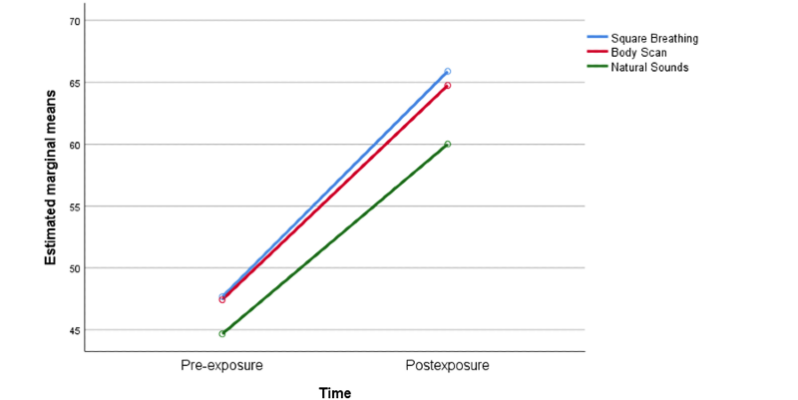
Perceived relaxation.

A significant moderate main effect for time was found (*F*[1,237]=121.5, partial *η*^2^=.34). Therefore, relaxation scores after the exposures were significantly higher than before listening to the audio clips (Figure 1). There was a nonsignificant effect of the group, indicating that the ratings from all three groups were similar (*F*[2,237]=1.15, *P*=.32, partial *η*^2^=.01). Thus, there was no overall difference in the scores of perceived relaxation between the square breathing, body scan, and natural sounds groups. Finally, results revealed a nonsignificant time x group interaction (*F*[2,237]=.32, *P*=.73, partial *η*^2^=.003).

**Figure 2:**
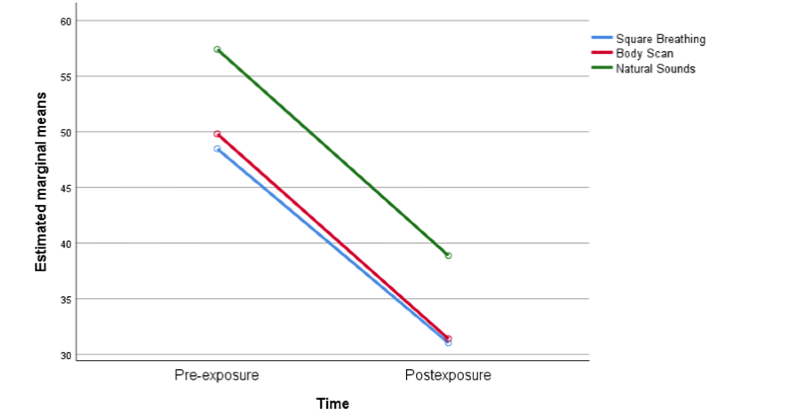
Psychomotor activation/stress.

A significant main effect of time was found (*F*[1,237]=153.5, partial *η*^2^=.39). Hence, perceived psychomotor activation/stress scores after the exposures were significantly lower than before participants listened to the audio clips and the effect was moderate (Figure 2). Results also showed a nonsignificant time x group interaction (*F*[2,237]=.06, *P*=.95, partial *η*^2^=.0001), while a small significant effect of the groups was found (*F*[2,237]=3.6, *P*=.03, partial *η*^2^=.03). Contrasts between groups and post hoc tests revealed that the guided audio clips (ie, square breathing, body scan) yielded a significantly lower level of perceived stress compared to the natural sounds audio clip.

Specifically, those who were in the square breathing condition were significantly (*P*=.04) less stressed than the participants in the natural sounds group and those who were in the body scan condition rated their perceived level of stress as lower than the participants in the natural sounds group (*P*=.03).

**Figure 3:**
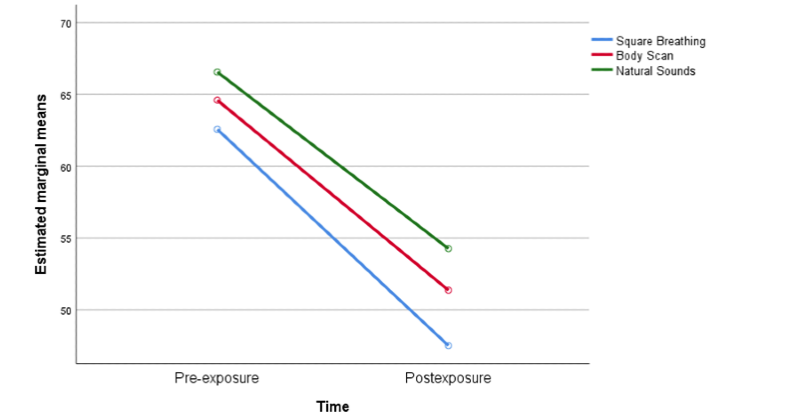
Thoughts related to COVID-19.

A significant main effect for time was found (*F*[1,237]=103.4, partial *η*^2^=.3). Therefore, the degree of fear of thoughts related to COVID-19 after the exposures was significantly lower than before exposure to the audio clips (Figure 3). There was a nonsignificant effect of the group, indicating that the ratings from all three groups were similar (*F*[2,237]=.95, *P*=.38, partial *η*^2^=.01). Thus, there was no overall difference in the scores of preoccupations related to COVID-19 between the square breathing, body scan, and natural sounds groups. Finally, results showed a nonsignificant time x group interaction (*F*[2,237]=.37, *P*=.69, partial *η*^2^=.003).

Overall, all three variables showed a positive improvement following the audio clips, while no significant differences between the groups emerged for perceived relaxation and for COVID-19–related preoccupation, while significant differences emerged between groups in perceived psychomotor activation/stress, where both the guided audio clips yielded a decrease in perceived stress compared to natural sounds.

### Emotional State (Valence, Arousal, and Dominance)

We performed a nonparametric analysis on the items of the SAM. To test if there was a difference in pre-post scores of the perceived emotional state, we ran, for each experimental group, the Wilcoxon signed-rank test on variables assessed before and after exposure to the audio clips and reported the ES in the form of Hodges-Lehmann ES.

For all groups, a significant positive improvement was observed on the dimensions of the pleasantness of the emotional state (valence) and perceived control over it (dominance), while a nonsignificant trend resulted for arousal.

With regard to the square breathing group, results showed a significant positive improvement in valence (T=640, *z*=3.16, *P*=.002; ES=0.5, 95% CI 0-1) and perceived dominance (T=477, *z*=3.12, *P*=.002; ES=0.5, 95% CI 0-1), while a nonsignificant trend was found for perceived arousal (T=707.5, *z*=1.84, *P*=.06; ES=0.5, 95% CI 0-1). For the body scan group, valence and dominance improved (T=1045, *z*=5.18, *P*<.001; ES=1, 95% CI 1-1.5 and T=393.5, *z*=2.88, *P*=.004; ES=0.5, 95% CI 0-1, respectively), while arousal did not (T=516, *z*=.25, *P*=.80; ES=0, 95% CI –5 to 5). Finally, the effects for the natural sounds group were T=943.5, *z*=3.70, *P*=.001; ES=0.5, 95% CI 0.5-1 for valence, T=516.5, *z*=2.18, *P*=.03; ES=0, 95% CI 0-0.5 for dominance, and T=457, *z*=.35, *P*=.728; ES=0, 95% CI 0-1 for arousal.

To assess between-groups differences, we performed the Kruskal-Wallis test as a global test on all three groups. None of the comparisons between the groups yielded significant differences; thus, we did not proceed with paired comparisons.

Specifically, valence yielded *χ*^2^_2_=4.6, *P*=.1 pre-exposure and *χ*^2^_2_=1.17, *P*=.56 postexposure, while arousal scores gave *χ*^2^_2_=2.56, *P*=.28 pre-exposure and *χ*^2^_2_=2.17, *P*=.34 postexposure, and analysis of dominance showed *χ*^2^_2_=0.18, *P*=.91 pre-exposure and *χ*^2^_2_=1.25, *P*=.54 postexposure.

### Role of Baseline Anxiety on Intervention Efficacy

To test if baseline levels of anxiety moderated the efficacy of the audio clips, we performed mixed ANOVA testing for moderation effects of the questionnaire scores for STAI-Y, ASI-3, and BVS with group and time. Correlations between questionnaires and postexposure scores are reported in Table 4.

Overall, there was a significant moderation effect between STAI-Y scores and time (*F*[1,234]=7.22, *P*=.008, partial *η*^2^=.03) on perceived relaxation and the same pattern was observed for the interaction with time (*F*[1,234]=4.4, *P*=.04, partial *η*^2^=.02) on psychomotor activation/stress, suggesting a higher efficacy for those participants who were less anxious at baseline.

In regard to the ASI-3, we found that there was a significant moderation effect between ASI-3 levels and time (*F*[1,234]=4.3, *P*=.04, partial *η*^2^=.02) on perceived relaxation, while a three-way interaction was found between group, time, and ASI-3 for the effect on perceived psychomotor activation/stress (*F*[1,234]=3.2, *P*=.04, partial *η*^2^=.03). For BVS scores, a significant moderation effect was found between BVS and time (*F*[1,228]=5.4, *P*=.02, partial *η*^2^=.02) on perceived relaxation. As for trait anxiety, these results suggested that there was less efficacy for those participants who reported higher body vigilance anxiety at baseline.

### Qualitative Results

Qualitative reports on subjective experiences and suggestions were examined to understand the participants’ subjective experience and to determine possible improvements and issues related to the proposed stimuli. Within the discussion, practical advice for future studies is given accordingly.

Participants were then asked to identify the pros and cons of the proposed web-based relaxation practices. Most of them (75/240) liked audio features such as the teacher’s recorded voice or the sound of water (if present). Participants also loved the sense of relaxation (55/240) or awareness (19/240) they achieved after the intervention. On the other hand, some people experienced boredom or annoyance due to the recorded audio.

Finally, a few participants also gave some suggestions for improving the proposed interventions; specifically, they would vary the length of the intervention and give more precise instructions during the practice.

## Discussion

### Principal Findings

People are facing the COVID-19 pandemic worldwide. The disease and its unknown long-term consequences have triggered an increase in stress levels and arousal. Moreover, the lockdown forced people into social isolation and prevented the implementation of in-person programs to target psychological issues. Noteworthy in our sample, which was composed of Italian citizens under social distancing restrictions, anxiety levels were slightly increased compared to normative data.

In such a situation, delivering helpful interventions to manage stress and anxiety becomes increasingly difficult. However, web-based interventions have been increasingly adopted in clinical practice for facilitating psychological assessment and enabling the delivery of the same treatment to many subjects concurrently [36].

This randomized study compared the effects of remotely delivered interventions using natural sounds, deep breathing, and meditation on perceived relaxation, psychomotor activation, level of preoccupation with COVID-19, and emotional state, as well as on people’s experiences with these techniques. Indeed, a comparison of natural sounds, respiration, and body scan meditation techniques delivered as web-based interventions was missing in the literature.

In accordance with our first prediction, results showed that all three techniques produced positive effects on perceived relaxation, stress, and preoccupation related to COVID-19. Specifically, we found that perceived relaxation levels, psychomotor activation/stress, and disturbing thoughts related to COVID-19 significantly improved after exposure to the three audio clips, with a moderate effect. Starting from this evidence, we concluded that the audio clips were effective in inducing a calmer psychological state. Our findings are consistent with the results of another study that aimed to reduce anxiety and depression in patients with COVID-19, which was a web-based intervention containing breath relaxation training, a mindfulness body scan, and behavioral techniques that significantly improved mood disturbance symptoms [37].

Based on between-groups differences, results showed significant differences only on perceived psychomotor activation/stress, where guided exercises (ie, square breathing and body scan) were more effective than the natural sounds audio clip. No other differences in efficacy between groups were observed. These results partially confirmed our second hypothesis, as we obtained enhanced efficacy only on the dimension of stress and not for perceived relaxation.

We concluded that for this brief web-based relaxation intervention, the three audio clips were effective at improving psychological adjustment. We speculated that, even if the audio clips involved different psychocognitive activities (ie, regulating breath frequencies, bringing awareness to body sensations, and simply listening to natural sounds), for brief relaxation experiences, these different processes did not have significant variances in efficacy. Thus, for the purpose of simple and brief relaxation interventions, all three can be effectively applied. Consistently, Jain and colleagues [38] conducted a randomized controlled trial on the differences among mindfulness meditation, relaxation training, and a waitlist control group in reducing distress; no statistical differences were found between the two treatment groups, even if both interventions significantly improved positive emotional state and reduced distress levels.

Furthermore, regarding differences between groups, we posit that the guided exercises yielded a greater effect than natural sounds for perceived stress because they ask users to bring their attention to body sensations and breathing frequency, which promotes a calm state. A systematic review of the importance of guidance during web-based interventions showed guided interventions are more effective than nonguided ones [39]. Such instructions may not change the degree of muscle contraction or body perception, but rather they can encourage a state of pleasant awareness, which is in line with the purpose of meditation practices. In fact, answering the final open-ended questions, participants reported that the audio clips allowed them to have a break and think about their breathing rhythm, thus regulating it. Others had the opportunity to observe their “inner world” becoming calmer. Moreover, audio features may vary the pleasantness of the audio clips; most of the participants reported positive feelings and a sense of relaxation in response to the guided body scan and square breathing interventions. Nevertheless, other participants did not like the tone of the voice and became bored, hyperactive, or upset.

In regard to participants’ emotional state, assessed through the SAM, we found that all the audio clips were effective in improving the valence (pleasure) of the emotional experience and the perceived dominance over it, while no significant results were found on the dimension of degree of arousal related to the emotional state. On the basis of these latter results, we speculated that the relaxing audio clips were more effective at enhancing the pleasantness and sense of control of one’s emotions, rather than the physical activation linked to such an emotional state.

### Limitations and Future Directions

This study has some limitations. First, it was based on single-session guided interventions. Thus, we cannot assess differences in the efficacy of more prolonged exposure to relaxation sessions and correct for the effect of training or habituation. Future studies might adjust the length of the audio clips in light of the participants’ reports and/or propose repeated exposure to the audio clips.

Second, in this study, we did not include a neutral control group and we were not able to assess the efficacy of the techniques employed in the audio clips compared to no treatment. We could only assess the differences between the three active groups. In addition, the lack of a neutral control group meant we could not compare the effect of simply remaining in a passive resting state for 7 minutes with the effect of the interventions we delivered.

Third, being a remote intervention, we could not control for participants listening to the audio clips. We inserted a self-report check at the end of the audio clips that asked if participants really listened to the audio clips, but we did not have objective measures of the behavior and degree of attention of participants while listening. Similarly, in this study we employed only self-reported measures; as our intervention was delivered in the context of social distancing, other approaches were not feasible to implement. Future studies with more objective measures and psychophysiological variables (such as skin conductance) would strengthen the evidence of the efficacy of these techniques.

Fourth, we did not perform intention-to-treat analysis on our sample because we could not analyze participants who stopped before the audio clips or during the audio clips, since they had completed only the preintervention questionnaire. Therefore, analyses were performed only on participants who also completed the postintervention questionnaire. However, we checked for the presence of differences in the main psychosocial variables (age, gender, the presence of a chronic disease, anxiety scores) between the initial sample and the final sample, and we found no significant differences.

Finally, considering the possibility of applying such interventions to a clinical population, future studies might further explore the relationship between trait anxiety and the efficacy of the techniques. In this study, we could not perform a complex moderation model, nor we could draw conclusions on how different levels and different types of anxiety might shape the efficacy of the techniques. Preliminary evidence points to the potential moderating effect of baseline anxiety on the ES of relaxation interventions.

### Conclusions

Despite these limitations, our study demonstrated that even a very brief online intervention that is based on this approach can contribute to a significant stress reduction. This study provides a novel insight that can orient the development of future low-cost web-based interventions to reduce preoccupation and anxiety in the general population. Future studies might also assess and compare the efficacy of these approaches in clinical protocols for patients with anxiety and hyperarousal.

## Appendix

Multimedia Appendix 1 Chronic diseases reported by the participants.

Multimedia Appendix 2 CONSORT-eHEALTH V1.6.2 checklist.

## Abbreviations

ANOVA: analysis of variance
ASI-3: Anxiety Sensitivity Index-3
BVS: Body Vigilance Scale
CONSORT: Consolidated Standards of Reporting Trials
ES: effect size
SAM: Self-Assessment Manikin
STAI-Y: State-Trait Anxiety Inventory form-trait subscale
VAS: Visual Analogue Scale
