# Supplementary material for: Web-Based Relaxation Intervention for Stress During Social Isolation: Randomized Controlled Trial
Source: JMIR Ment Health. 2020 Dec 3;7(12):e22757. doi: 10.2196/22757 (PMC8080491; doi:10.2196/22757)
Supplement: Multimedia Appendix 1 [file mental_v7i12e22757_app1.docx]

Appendix A – *Chronic diseases reported by the participants (N= 82).*

| **Chronic diseases** | **Frequency (%)** |
| --- | --- |
| Autoimmune | 43 (52.4) |
| Cancer | 3 (3.7) |
| Cardiovascular | 5 (6.1) |
| Gastrointestinal | 3 (3.7) |
| Metabolic dysfunction | 4 (4.9) |
| Multiple diseases | 13 (15.9) |
| Neurological | 4 (4.9) |
| Psychiatric | 1 (1.2) |
| Respiratory | 1 (1.2) |
| Musculoskeletal | 2 (2.4) |
| Sensory disease | 2 (2.4) |
| Total | 82 (100) |

*Note: chronic diseases were categorised in 11 groups. Those who declared more than one chronic condition were grouped in the ‘multiple’ category.*
