# Supplementary material for: Web-Based Relaxation Intervention for Stress During Social Isolation: Randomized Controlled Trial
Source: JMIR Ment Health. 2020 Dec 3;7(12):e22757. doi: 10.2196/22757 (PMC8080491; doi:10.2196/22757)
Supplement: Multimedia Appendix 2 [file mental_v7i12e22757_app2.pdf]

CONSORT-EHEALTH Checklist V1.6.2 Report	Manuscript Number	22757

(based on CONSORT-EHEALTH V1.6), available at [http://tinyurl.com/consort-ehealth-v1-6].

Date completed

7/23/2020 4:51:43

by

Silvia Francesca Maria Pizzoli

Remote relaxation interventions to face stress during social isolation: a randomized controlled trial

TITLE

1a-i) Identify the mode of delivery in the title

"Remote relaxation interventions to face stress during social isolation: a randomized controlled trial"

1a-ii) Non-web-based components or important co-interventions in title

There are no important co-intervention to report

1a-iii) Primary condition or target group in the title

The title reports 'during social isolation', as the study was conducted in the period of social restrictions

ABSTRACT

1b-i) Key features/functionalities/components of the intervention and comparator in the METHODS section of the ABSTRACT

The abstract contains information on the interventions and their technical features ("Participants were randomly assigned to one of three experimental conditions. Each condition was characterized by a single on-line session of guided Square Breathing exercise, guided Body Scan exercise, or Natural Sounds. The participants listened to one of the fully automated audio clips for 7 minutes and pre-post completed self-assessed scales on perceived relaxation, psychomotor activation, level of preoccupation associated with COVID-19 and emotional state. At the end of the session, qualitative reports on subjective experience were also collected.").

1b-ii) Level of human involvement in the METHODS section of the ABSTRACT

Yes it does ("The participants listened to one of the fully automated audio clips for 7 minutes and pre-post completed self-assessed scales on perceived relaxation, psychomotor activation, level of preoccupation associated with COVID-19 and emotional state. At the end of the session, qualitative reports on subjective experience were also collected.")

1b-iii) Open vs. closed, web-based (self-assessment) vs. face-to-face assessments in the METHODS section of the ABSTRACT

"Each condition was characterized by a single on-line session".. "The participants listened to one of the fully automated audio clips for 7 minutes and pre-post completed self-assessed scales "

1b-iv) RESULTS section in abstract must contain use data

"Overall, 294 participants completed more than half of the survey and 240 completed the entire survey."

1b-v) CONCLUSIONS/DISCUSSION in abstract for negative trials

INTRODUCTION

2a-i) Problem and the type of system/solution

"Applying these interventions to people who are forced into a mandatory social isolation may contribute to become more aware of their mind-body condition and reduce negative affects.
We must add that, exactly because of the restrictions due to COVID-19, face-to-face interventions were not applicable, while internet-based interventions represented a good opportunity. The usefulness of remote intervention is also supported by studies showing that online relaxation interventions reported significant results as well as in-person interventions [19,20]. However, a comparison among natural sounds, respiration and body scan meditation techniques in internet-based interventions is still missing in the literature."

2a-ii) Scientific background, rationale: What is known about the (type of) system

"Applying these interventions to people who are forced into a mandatory social isolation may contribute to become more aware of their mind-body condition and reduce negative affects.
We must add that, exactly because of the restrictions due to COVID-19, face-to-face interventions were not applicable, while internet-based interventions represented a good opportunity. The usefulness of remote intervention is also supported by studies showing that online relaxation interventions reported significant results as well as in-person interventions [19,20]. However, a comparison among natural sounds, respiration and body scan meditation techniques in internet-based interventions is still missing in the literature.
"

Does your paper address CONSORT subitem 2b?

"In the present study, we aimed to test and compare the efficacy of three remote interventions, respectively based on natural sounds, breathing regulation and body scan, to assess which intervention was the most effective for the target population. For this purpose, we tested the difference in the efficacy of three audio clips corresponding to the three relaxation practices (Square Breathing exercise, guided Body Scan exercise, and Natural Sounds) in stress reduction. Specifically, we expected to find: 1) a decrease in the levels of psychomotor activation/stress and of preoccupation for COVID-19, as well as enhanced levels of relaxation and emotional state after the exposure of all the audio clips; 2) guided techniques (Square Breathing and Body Scan) to have a greater effect on the above-mentioned dimensions, compared to Natural Sounds."

METHODS

3a) CONSORT: Description of trial design (such as parallel, factorial) including allocation ratio

"The participation in the study consisted of three main parts: (a) a short questionnaire containing socio-demographics, baseline anxiety evaluation (trait anxiety, anxiety for physical sensations, body vigilance) information and pre-intervention evaluation, (b) the listening of a 7 minutes audio-clip, (c) the post-intervention evaluation. The estimated time for participating in the study (completing the three parts) ranged from 12 to 17 minutes.".. "After completing questionnaires, participants were randomly assigned to one of the three experimental groups via the randomization procedure within Qualtrics. The randomization option was set to enrol the same number of subjects for each condition. "

3b) CONSORT: Important changes to methods after trial commencement (such as eligibility criteria), with reasons

There were no changes after trial commencement.

3b-i) Bug fixes, Downtimes, Content Changes

4a) CONSORT: Eligibility criteria for participants

"The eligibility criteria to take part in the study were: 1) being older than 18 years old, 2) being a proficient Italian speaker, 3) not suffering from any impairment affecting auditive abilities, and 4) having an appropriate familiarity with computer literacy. Before taking part in the study, participants were asked to read and to complete an online consent form. "

4a-i) Computer / Internet literacy

4a-ii) Open vs. closed, web-based vs. face-to-face assessments:

"During the first week of May 2020, the invitation to take part in the study was published in Italian social media webpages (specifically: WhatsApp, Facebook, LinkedIn, and Instagram). The readers were informed about the general aim of the study and that the study was conducted by researchers from the University of Milan. Potential participants were encouraged both to take part in the study and to share the invitation with their acquaintances. The invitation contained a link to the Qualtrics platform, where a more detailed description was available."

4a-iii) Information giving during recruitment

4b) CONSORT: Settings and locations where the data were collected

"The invitation contained a link to the Qualtrics platform, where a more detailed description was available."

4b-i) Report if outcomes were (self-)assessed through online questionnaires

"The participation in the study consisted of three main parts: (a) a short questionnaire containing socio-demographics, baseline anxiety evaluation (trait anxiety, anxiety for physical sensations, body vigilance) information and pre-intervention evaluation "..."After completing the sociodemographic form, participants were asked to report if they suffered from a chronic disease and how much the disease impacted their (perceived) vulnerability to COVID-19. Participants were asked to report their working situation, recent changes in occupational status due to COVID-19 restrictions, and if they had prior experience with relaxation techniques.
Then, participants were asked to complete 3 self-assessed questionnaires aimed at measuring the current level of anxiety (trait anxiety), the tendency of being worried about physical signals and sensations (anxiety for physical sensations), and the degree of attention paid to bodily feelings (body vigilance). For the assessment of these aspects, the following self-reported scales were used.."

4b-ii) Report how institutional affiliations are displayed

"The readers were informed about the general aim of the study and that the study was conducted by researchers from the University of Milan. "

5) CONSORT: Describe the interventions for each group with sufficient details to allow replication, including how and when they were actually administered

5-i) Mention names, credential, affiliations of the developers, sponsors, and owners

5-ii) Describe the history/development process

**5-iii) Revisions and updating**		
**5-iv) Quality assurance methods**		
**5-v) Ensure replicability by publishing the source code, and/or providing screenshots/screen-capture video, and/or providing flowcharts of the algorithms used**		
**5-vi) Digital preservation**		
**5-vii) Access**		
"the invitation to take part in the study was published in Italian social media webpages (specifically: WhatsApp, Facebook, LinkedIn, and Instagram). The readers were informed about the general aim of the study and that the study was conducted by researchers from the University of Milan. Potential participants were encouraged both to take part in the study and to share the invitation with their acquaintances. The invitation contained a link to the Qualtrics platform, where a more detailed description was available."		
**5-viii) Mode of delivery, features/functionalities/components of the intervention and comparator, and the theoretical framework**		
"The participation in the study consisted of three main parts: (a) a short questionnaire containing socio-demographics, baseline anxiety evaluation (trait anxiety, anxiety for physical sensations, body vigilance) information and pre-intervention evaluation, (b) the listening of a 7 minutes audio-clip, (c) the post-intervention evaluation. The estimated time for participating in the study (completing the three parts) ranged from 12 to 17 minutes."..."In each experimental condition, participants received a 7-minutes audio aimed at promoting a state of awareness and relaxation. In the first experimental condition (i.e. Square Breathing), participants heard a recorded voice guiding the regulation of breathing frequencies with the aim of making every breathing act (inhalation, hold breath, exhalation, hold breath) last the same time (4 seconds). In the second experimental condition, an audio with a voice which guided attention through every part of the body was presented and gently requested the listener to feel tensions, unpleasant feelings and to let them go (i.e. Body Scan). Both tracks were recorded by a trained mindfulness and Yoga expert in collaboration with a psychotherapist and were pre-tested on 4 subjects to assess the easiness of the exercise and the perceived effectiveness. In the latter condition (i.e. Natural Sounds) participants were presented by a pre-recorded audio with natural sounds. All the audio clips were preceded by instructions on the place and the recommended body position for the exercises."		
**5-ix) Describe use parameters**		
**5-x) Clarify the level of human involvement**		
**5-xi) Report any prompts/reminders used**		
The intervention was a single session one. No reminders or prompts were used.		
**5-xii) Describe any co-interventions (incl. training/support)**		
There no co-interventions.		
**6a) CONSORT: Completely defined pre-specified primary and secondary outcome measures, including how and when they were assessed**		
"As pre-post measures, before and after the audio stimuli, subjects were asked to self-rate perceived relaxation level, perceived stress and psychomotor activation degree, how much they felt concerned about COVID-19, and to rate 3 specific features of their emotional state. Specifically, participants were requested to rate on a 3 Visual Analogue Scale (VAS) (0= not at all-10=completely) how much they felt relaxed, stressed/activated and how much thoughts related to COVID-19 scared them; furthermore, they completed the Self-Assessment Manikin (SAM) [31] for emotional states, which is a three-items visual scale (valence, intensity/arousal, dominance) commonly used to quantify properties of the felt overall emotional state on 1 to 5 scales of images. To check if participants really listened to the audio clips, they were asked immediately after the audio if they heard the entire trace, a part of it or no part.<br>Finally, all the participants were asked to describe their personal experience and to provide suggestions for future changes. Specifically, participants were requested to write a short paragraph answering two open-ended questions about their personal experience with the exercise (i.e. what they liked and what they would have changed)."		
**6a-i) Online questionnaires: describe if they were validated for online use and apply CHERRIES items to describe how the questionnaires were designed/deployed**		
**6a-ii) Describe whether and how "use" (including intensity of use/dosage) was defined/measured/monitored**		
**6a-iii) Describe whether, how, and when qualitative feedback from participants was obtained**		
**6b) CONSORT: Any changes to trial outcomes after the trial commenced, with reasons**		
"The invitation contained a link to the Qualtrics platform, where a more detailed description was available."		
**7a) CONSORT: How sample size was determined**		
**7a-i) Describe whether and how expected attrition was taken into account when calculating the sample size**		
A study protocol with sample size computation was previous published.		
**7b) CONSORT: When applicable, explanation of any interim analyses and stopping guidelines**		
"As pre-post measures, before and after the audio stimuli, subjects were asked to self-rate perceived relaxation level, perceived stress and psychomotor activation degree, how much they felt concerned about COVID-19, and to rate 3 specific features of their emotional state. Specifically, participants were requested to rate on a 3 Visual Analogue Scale (VAS) (0= not at all-10=completely) how much they felt relaxed, stressed/activated and how much thoughts related to COVID-19 scared them; furthermore, they completed the Self-Assessment Manikin (SAM) [31] for emotional states, which is a three-items visual scale (valence, intensity/arousal, dominance) commonly used to quantify properties of the felt overall emotional state on 1 to 5 scales of images. To check if participants really listened to the audio clips, they were asked immediately after the audio if they heard the entire trace, a part of it or no part.<br>Finally, all the participants were asked to describe their personal experience and to provide suggestions for future changes. Specifically, participants were requested to write a short paragraph answering two open-ended questions about their personal experience with the exercise (i.e. what they liked and what they would have changed)."		
**8a) CONSORT: Method used to generate the random allocation sequence**		
"After completing questionnaires, participants were randomly assigned to one of the three experimental groups via the randomization procedure within Qualtrics. The randomization option was set to enrol the same number of subjects for each condition. "		
**8b) CONSORT: Type of randomisation; details of any restriction (such as blocking and block size)**		
"After completing questionnaires, participants were randomly assigned to one of the three experimental groups via the randomization procedure within Qualtrics. The randomization option was set to enrol the same number of subjects for each condition. "		
**9) CONSORT: Mechanism used to implement the random allocation sequence (such as sequentially numbered containers), describing any steps taken to conceal the sequence until interventions were assigned**		
"After completing questionnaires, participants were randomly assigned to one of the three experimental groups via the randomization procedure within Qualtrics. The randomization option was set to enrol the same number of subjects for each condition. "		
**10) CONSORT: Who generated the random allocation sequence, who enrolled participants, and who assigned participants to interventions**		
"After completing questionnaires, participants were randomly assigned to one of the three experimental groups via the randomization procedure within Qualtrics. The randomization option was set to enrol the same number of subjects for each condition. "		
**11a) CONSORT: Blinding - If done, who was blinded after assignment to interventions (for example, participants, care providers, those assessing outcomes) and how**		
**11a-i) Specify who was blinded, and who wasn't**		
Subject were not blind to the condition, while the researchers who run the analyses and who assessed the subjective reports were blind.		
**11a-ii) Discuss e.g., whether participants knew which intervention was the "intervention of interest" and which one was the "comparator"**		
**11b) CONSORT: If relevant, description of the similarity of interventions**		
It is not relevant.		
**12a) CONSORT: Statistical methods used to compare groups for primary and secondary outcomes**		
"To test the difference in efficacy between audio clips, a one-way ANOVA on gain relaxation scores, with 3 groups and fixed effects, with no interaction was performed, without considering subjects who stopped before the randomized exposure to the audio clips. Furthermore, to assess group and time effect and their interaction, a 2 (Time) x 3 (Groups) mixed-model ANOVA was performed for perceived relaxation scores, perceived stress/activation and preoccupations related to COVID-19. We also performed nonparametric analysis on the items of SAM, as statistical assumptions for parametrical analysis were violated. In this case, we reported the effect size in the form of Hodges and Lehmann effect size."		
**12a-i) Imputation techniques to deal with attrition / missing values**		
"294 gave written informed consent and completed more than the 75% of the survey (meaning that they complete the initial questionnaire and were randomized into the three conditions), 240 completed the entire survey and stated at the check that they listened to the audio clips: 77 in Square Breathing group, 76 in the Body Scan group and 87 in the Natural Sounds one. ".<br>Participant who declared they did not listen or heard the audio clips were not included in the analysis.		
**12b) CONSORT: Methods for additional analyses, such as subgroup analyses and adjusted analyses**		

"To test the difference in efficacy between audio clips, a one-way ANOVA on gain relaxation scores, with 3 groups and fixed effects, with no interaction was performed, without considering subjects who stopped before the randomized exposure to the audio clips. Furthermore, to assess group and time effect and their interaction, a 2 (Time) x 3 (Groups) mixed-model ANOVA was performed for perceived relaxation scores, perceived stress/activation and preoccupations related to COVID-19. We also performed nonparametric analysis on the items of SAM, as statistical assumptions for parametrical analysis were violated. In this case, we reported the effect size in the form of Hodges and Lehmann effect size.<br>Explorative analyses on the possible role of trait anxiety, anxiety for physical sensations, body vigilance in moderating the effect of the audio clips were also carried out. "		
RESULTS		
**13a) CONSORT: For each group, the numbers of participants who were randomly assigned, received intended treatment, and were analysed for the primary outcome**		
"240 completed the entire survey and stated at the check that they listened to the audio clips: 77 in Square Breathing group, 76 in the Body Scan group and 87 in the Natural Sounds one. ".<br>Tables and results report the number of analyzed subjects.		
**13b) CONSORT: For each group, losses and exclusions after randomisation, together with reasons**		
"294 gave written informed consent and completed more than the 75% of the survey (meaning that they complete the initial questionnaire and were randomized into the three conditions), 240 completed the entire survey and stated at the check that they listened to the audio clips: 77 in Square Breathing group, 76 in the Body Scan group and 87 in the Natural Sounds one. ".<br>Other losses were due to missing data.		
**13b-i) Attrition diagram**		
**14a) CONSORT: Dates defining the periods of recruitment and follow-up**		
"During the first week of May 2020, the invitation to take part in the study was published in Italian social media webpages (specifically: WhatsApp, Facebook, LinkedIn, and Instagram). "		
**14a-i) Indicate if critical “secular events” fell into the study period**		
**14b) CONSORT: Why the trial ended or was stopped (early)**		
The trial ended with the end of social restrictions.		
**15) CONSORT: A table showing baseline demographic and clinical characteristics for each group**		
Baseline measures and demographic features are reported for all the assessed variables. "Table 1: Descriptive statistics of the sample" .. "Table 2: Descriptive statistics of the total scores of the questionnaire scores and correlations between total scores".. "Descriptive statistics of all the assessed pre-post variables for each group are depicted in Table 3. "		
**15-i) Report demographics associated with digital divide issues**		
"Table 1: Descriptive statistics of the sample"P		
**16a) CONSORT: For each group, number of participants (denominator) included in each analysis and whether the analysis was by original assigned groups**		
**16-i) Report multiple “denominators” and provide definitions**		
Participants were analysed by original assigned groups.<br>The trial employed a single session intervention, there were no multiple uses of the exercises.<br>Effect sizes are reported.		
**16-ii) Primary analysis should be intent-to-treat**		
**17a) CONSORT: For each primary and secondary outcome, results for each group, and the estimated effect size and its precision (such as 95% confidence interval)**		
For each primary and secondary outcomes results for each group were presented (example: primary outcomes: "A significant main effect for Time was found, F(1,237) = 103.4, partial η^2 = .3. Therefore, the degree of fear for thoughts related to COVID-19 after the exposures were significantly lower than before the exposure to the audio clips. There was a non-significant effect of the group, indicating that the ratings from all the three groups were similar, F(2, 237) = .95, P =.38, partial η^2 = .01 Thus, there was no overall difference in the scores of preoccupations related to the COVID-19 between Square Breathing, Body Scan and Natural Sounds. Finally, results showed a non-significant Time x Group interaction, F(2,237) = .37, P = .69, partial η^2 = .003. "; secondary outcomes: "For what concern the Square Breathing group, results showed a significant positive improvement in the valence (T= 640, z= 3.16, P =.002, e.s.= .5, 95%CI [0,1]) and in perceived dominance (T= 477, z= 3.12, P =.002, e.s. = .5, 95%CI [0,1]), while a non-significant trend was found in perceived arousal (T= 707.5, z= 1.84, P =.065, e.s. = .05, 95%CI [0,1]). ")		
**17a-i) Presentation of process outcomes such as metrics of use and intensity of use**		
**17b) CONSORT: For binary outcomes, presentation of both absolute and relative effect sizes is recommended**		
There were no binary outcomes.		
**18) CONSORT: Results of any other analyses performed, including subgroup analyses and adjusted analyses, distinguishing pre-specified from exploratory**		
"Explorative analyses on the possible role of trait anxiety, anxiety for physical sensations, body vigilance in moderating the effect of the audio clips were also carried out. "<br>"To test if baseline levels of anxiety moderated the efficacy of the audio clips, we performed Mixed ANOVA testing for moderation effects of the questionnaire scores STAI-Y, ASI-3, BVS with group and time. Correlation between questionnaires and post-exposure scores are reported in Table 3."		
**18-i) Subgroup analysis of comparing only users**		
**19) CONSORT: All important harms or unintended effects in each group**		
There were no unintended effects of harms in the experimental groups.		
**19-i) Include privacy breaches, technical problems**		
**19-ii) Include qualitative feedback from participants or observations from staff/researchers**		
DISCUSSION		
**20) CONSORT: Trial limitations, addressing sources of potential bias, imprecision, multiplicity of analyses**		
**20-i) Typical limitations in ehealth trials**		
"The study presents some limitations. First, it was based on single-session guided interventions. Thus, we cannot assess differences in the efficacy of more prolonged exposure to relaxation sessions and correct for the effect of training or habituation. However, our study demonstrated that even a very brief online intervention that is based on this approach can contribute to a significant stress reduction. Future studies might adjust the length of the audio clips also in the light of the participants’ reports.<br>A second limitation of the present study is that we employed only self-reported measures. Indeed, as our intervention was delivered in a context of social distancing, other approaches were impossible to implement. However, future studies with more objective measures and psychophysiological variables (such as skin conductance), would strengthen the evidences of the efficacy of these techniques.<br>Finally, considering the possibility of applying such intervention also to a clinical population, future studies might also deepen the relationship between baseline and trait anxiety and the efficacy of the techniques. In the present study, we could not perform complex moderation model, nor we could draw conclusions on how different levels and different type of anxiety might shape the efficacy of the techniques. Partial preliminary evidences from moderation effects seem to point out a moderation role of anxiety. "		
**21) CONSORT: Generalisability (external validity, applicability) of the trial findings**		
**21-i) Generalizability to other populations**		
**21-ii) Discuss if there were elements in the RCT that would be different in a routine application setting**		
**22) CONSORT: Interpretation consistent with results, balancing benefits and harms, and considering other relevant evidence**		
**22-i) Restate study questions and summarize the answers suggested by the data, starting with primary outcomes and process outcomes (use)**		
"The present randomized study provided data on the effectiveness of remotely delivered interventions with natural sounds, deep breathing and meditation practice in perceived relaxation, psychomotor activation, level of preoccupation associated with COVID-19 and emotional state, as well as on people’s experiences with these techniques. It also shed light on if and to what extent one of these exercises provides greater benefits compared to the others. Indeed, a comparison among natural sounds, respiration and body scan meditation techniques in internet-based interventions was missing in the literature.<br>In accordance with our first prediction, results showed that all the three techniques were effective in producing positive effects on the variables assessing perceived relaxation, stress and preoccupation related to COVID-19. Specifically, we found that perceived relaxation levels, psychomotor activation/stress and disturbing thoughts related to COVID-19 significantly improved after the exposure to the three audio clips with a moderate effect. Considering these evidences, we concluded that the audio clips were effective in inducing a calmer psychological state. Our findings are consistent with the obtained results of another study aiming at reducing anxiety and depression in patients with COVID-19: a remote intervention containing breath relaxation training, mindfulness body scan and behavioral techniques significantly improved mood disturbance symptoms [34]."		
**22-ii) Highlight unanswered new questions, suggest future research**		

Other information		
**23) CONSORT: Registration number and name of trial registry**		
International Registered Report Identifier (IRRID): PRR1-10.2196/19236 [22].		
**24) CONSORT: Where the full trial protocol can be accessed, if available**		
Pizzoli SMF, Marzorati C, Mazzoni D, Pravettoni G. An Internet-Based Intervention to Alleviate Stress During Social Isolation With Guided Relaxation and Meditation: Protocol for a Randomized Controlled Trial. JMIR Res Protoc [Internet] JMIR Publications Inc.; 2020 Jun 17;9(6):e19236. [doi: 10.2196/19236]		
**25) CONSORT: Sources of funding and other support (such as supply of drugs), role of funders**		
Authors disclosed no conflict of interest and no funding sources.		
**X26-i) Comment on ethics committee approval**		
"Participation in the study was voluntary and participants were informed that they could withdraw from the study at each moment. The research protocol followed ... the principles stated in the Declaration of Helsinki (59th WMA General Assembly, Seoul, 2008). The research protocol was approved by the Ethical Committee of the first author's University on April 30th, 2020 "		
**x26-ii) Outline informed consent procedures**		
**X26-iii) Safety and security procedures**		
**X27-i) State the relation of the study team towards the system being evaluated**		
